# Co-Amorphous Simvastatin-Nifedipine with Enhanced Solubility for Possible Use in Combination Therapy of Hypertension and Hypercholesterolemia

**DOI:** 10.3390/molecules23092161

**Published:** 2018-08-28

**Authors:** Cecilia Martínez-Jiménez, Jorge Cruz-Angeles, Marcelo Videa, Luz María Martínez

**Affiliations:** School of Engineering and Sciences, Department of Sciences—Chemistry and Nanotechnology, Tecnologico de Monterrey, Campus Monterrey, Av. Eugenio Garza Sada 2501 Sur. Monterrey N.L., Monterrey 64849, Mexico; a01139552@itesm.mx (C.M.-J.); a00800376@itesm.mx (J.C.-A.); mvidea@itesm.mx (M.V.)

**Keywords:** solubility, Nifedipine, Simvastatin, co-amorphous, amorphous drug, hypertension, hypercholesterolemia

## Abstract

The high index of simultaneous incidence of hypertension and hypercholesterolemia in the population of many countries demands the preparation of more efficient drugs. Therefore, there is a significant area of opportunity to provide as many alternatives as possible to treat these illnesses. Taking advantage of the solubility enhancement that can be achieved when an active pharmaceutical ingredient (API) is obtained and stabilized in its amorphous state, in the present work, new drug-drug co-amorphous formulations (Simvastatin SIM- Nifedipine NIF) with enhanced solubility and stability were prepared and characterized. Results show that the co-amorphous system (molar ratio 1:1) is more soluble than the pure commercial APIs studied separately. Aqueous dissolution profiles showed increments of solubility of 3.7 and 1.7 times for SIM and NIF, correspondingly, in the co-amorphous system. The new co-amorphous formulations, monitored in time, (molar fractions 0.3, 0.5 and 0.7 of SIM) remained stable in the amorphous state for more than one year when stored at room temperature and did not show any signs of crystallization when re-heating. Inspection on the remainder of a sample after six hours of dissolution showed no recrystallization, confirming the stability of co-amorphous system. The enhanced solubility of the co-amorphous formulations makes them promising for simultaneously targeting of hypertension and hypercholesterolemia through combination therapy.

## 1. Introduction

Hypertension and hypercholesterolemia (HT-HCL) are present simultaneously in the population of many countries. Studies have reported that, in Western Europe, United States, and Latin American countries, the prevalence of concomitant hypertension and hypercholesterolemia in adult population is in the range of 15 to 30% [1,2,3,4]. Table 1 shows a list of oral administrated drugs most commonly used to treat these diseases. Even when a wide range of active pharmaceutical ingredients (APIs) are available to treat these illnesses, the high simultaneous incidence of HT-HCL demands more efficient drugs and as many alternatives as possible to treat these particular medical problems, including combined therapy. This suggests that there is a significant area of opportunity for the improvement of current pharmaceutical formulations. As it can be seen in Table 1, most of the APIs currently prescribed to treat HT and HCL correspond to type II drugs (which have high permeability but low solubility); therefore new formulations with enhanced solubility could be an alternative to more efficiently treat health problems related with concomitant hypertension and hypercholesterolemia.

Formulation of active pharmaceutical ingredients (APIs) in amorphous state has been introduced in the last two decades as an alternative to solve their low solubility problem. It has been demonstrated that the amorphous state of an API has higher solubility and higher dissolution rate than its crystalline state, which is the common structural state of commercial drugs [13,14,15,16]. The enhancement that an amorphous drug presents in the release of a larger amount of molecules into a solution (which can be explained in terms of the ease to overcome intermolecular forces in an amorphous state compared to a crystal lattice) is also accompanied of a thermodynamic instability, since substances in the amorphous state are metastable and tend to recrystallize. An effective strategy to stabilize the amorphous state of an API is the preparation of amorphous binary formulations (co-amorphous systems), which is based on the addition of a second component that breaks the crystalline order of the former, allowing the formation of new intermolecular interactions that avoid recrystallization. Co-amorphous systems in which both components act simultaneously as active ingredients and stabilizing agents have gained interest in pharmaceutical research [16,17,18]. This strategy presents the opportunity to prepare formulations for complementary or combination therapies: releasing both drugs in a synchronic way to treat two different illnesses or to synergize the effect of two APIs used to treat a particular medical problem. After a literature review, and to the best of the authors’ knowledge, just a few published works were found in which the use of combination therapy relate to the treatment of HT-HCL. Furthermore, these studies are limited to the evaluation of the dissolution kinetics or the stability of amorphous state, but not both, indicating the need of a complete description of this kind of formulations [19,20].

The present work introduces a new co-amorphous system with two APIs intended for combination therapy focusing on treating hypertension and hypercholesterolemia, whose thermal and structural stability, as well as its enhanced solubility, were evaluated. The APIs selected for the study were Simvastatin (SIM) and Nifedipine (NIF). SIM is a cholesterol-lowering agent that belongs to the class of medication referred to as statins, widely used to treat hypercholesterolemia [21]. NIF is a calcium channel blocker for the treatment of cardiovascular diseases, such as hypertension [22]. Chen et al. [23] reported a protection effect of the presence of crystalline SIM and NIF on human umbilical vein endothelial cell (HUVEC) cultures. The study demonstrates that there is a synergistic effect when cells are grown in presence of both drugs resulting from an improved expression of mRNA and the protein nitric oxide synthase (eNOS), which increases the secretion of NO by inhibiting intracellular ROS generation, enhancing the protective effect of the two drugs on endothelial cells. It is important to mention that these individual drugs are commonly used in their crystalline form in health services to treat separately hypertension or hypercholesterolemia and both of them are classified as Class II drugs (low solubility; high bioavailability) according to the Biopharmaceutical Classification System (BCS).

## 2. Materials and Methods

### 2.1. Materials

**Figure 1:**
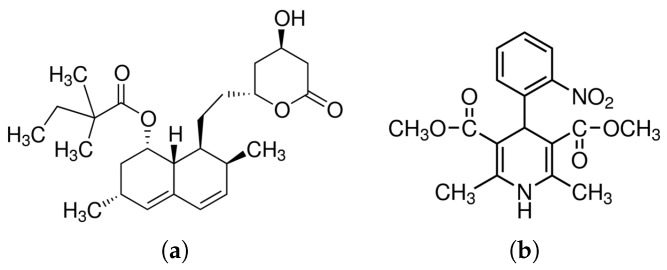
Chemical structures of active pharmaceutical ingredients (**a**) Simvastatin SIM (cholesterol lowering agent) and (**b**) Nifedipine NIF (calcium channel blocker used to treat hypertension).

Simvastatin ($C_{25}H_{38}O_{5}$, pharmaceutical secondary standard, MW: 418.57 g/mol), and Nifedipine ($C_{17}H_{18}N_{2}O_{6}$, ≥ 98% HPLC, MW: 346.33 g/mol) were purchased from Sigma Aldrich (St. Louis, MO, USA). The structures are shown in Figure 1. USP Phosphate buffer solution *p*H 6.8 was prepared using monobasic potassium phosphate (J.T. Baker, ACS reagent, Columbus, OH, USA) and sodium hydroxide pellets (Macron, AR (ACS), Center Valley, PA, USA) dissolved with HPLC grade deionized water. HPLC grade acetonitrile was purchased from J.T. Baker (Columbus, OH, USA).

### 2.2. Sample Preparation

Crystalline mixtures of the APIs (SIM-NIF) were weighted according to the corresponding molar ratio and gently mixed in a mortar for 1 min.

Amorphous pure APIs and co-amorphous formulations were prepared by melt-quenching: pure crystalline APIs or binary mixtures of different molar fractions (x${}_{\mathrm{SIM}}$ = 0.1, 0.2, 0.3, 0.5, 0.7, 0.8 and 0.9) were placed in an aluminum pan inside an oven and held at 180 ${}^{\circ }$C for one minute for the binary mixture, 165 ${}^{\circ }$C for SIM and 190 ${}^{\circ }$C for NIF. Once molten, the samples were removed from the oven and left to cool at room temperature.

### 2.3. Thermal Characterization

Thermal characterization was performed using a DSC–Pyris Diamond (PerkinElmer, Waltham, MA, USA) differential scanning calorimeter. Melting temperature of Indium (#LA94121V Perkin Elmer) served for calibration purposes. Thermal analysis was performed by placing 5–9 mg of the samples inside 50 μL aluminum pans (Perkin Elmer). In order to identify melting temperatures, samples were heated from 30 ${}^{\circ }$C to 180 ${}^{\circ }$C, the heating rate was 10 ${}^{\circ }$C/min under a constant nitrogen flow. Once molten, samples were amorphized in situ by cooling them from 180 ${}^{\circ }$C to −5${}^{\circ }$C at a rate of 70 ${}^{\circ }$C/min. After amorphization, a second thermal analysis was performed to determine their glass transition temperatures (Tg). Amorphous samples were stored in a desiccator at room temperature and measured after one year to determine the stability of the amorphous state calorimetrically.

### 2.4. Study of Storage Stability by XRD

Stability of the samples in their amorphous form was monitored using a Powder X-ray Diffractometer (PXRD) Rigaku Miniflex 600 (Tokyo, Japan). Measurements were performed with a source voltage of 30 kV and 15 mA using a Cu cathode (Kα) as a source. Samples were measured from 5${}^{\circ }$ to 37${}^{\circ }$ with a step size of 0.05${}^{\circ }$ and a speed of 2${}^{\circ }$/min for crystalline samples and 5${}^{\circ }$/min for amorphous samples. The amorphous samples for PXRD analysis were prepared by melting crystalline samples directly on a 22 × 22 mm cover glass used as a sample holder; this procedure allowed having amorphous samples permanently mounted on the cell holder on a fixed position. Samples were stored in a desiccator at room temperature and were continuously monitored as a function of storage time.

Also, the remaining undissolved excess of the material from a tablet of a SIM–NIF 1:1 after dissolution profile was subjected to XRD analysis to investigate the possible occurrence of recrystallization during the dissolution process.

### 2.5. Dissolution Profiles

For the study of the dissolution profiles, amorphous or crystalline samples were compacted into tablets (140 mg total for co-amorphous formulations) using a hydraulic press, applying a compression of 60 kg/cm${}^{2}$ for 1 min. Each tablet was inserted into a stainless steel basket, which was then placed in a water-jacketed glass vessel with 200 mL of the dissolution medium at 37 ${}^{\circ }$C and with magnetic stirring at 350 rpm. Samples were tested for solubility in two different media: HPLC grade deionized water and USP phosphate buffer pH 6.8. At predetermined times during 24 h, a 1 mL aliquot was withdrawn using a syringe equipped with a 0.2 μm nylon filter and immediately replaced with 1 mL of the corresponding dissolution medium. Experiments were performed in triplicate and analyzed by HPLC as described below. Experiments with samples containing NIF were performed protected from light due to its sensitivity [24].

### 2.6. Dissolution Kinetics Analysis by High Performance Liquid Chromatography (HPLC)

Simultaneous quantification of SIM and NIF was performed using a high-pressure liquid chromatography (HPLC) reverse-phase method. HPLC method was developed based on a previous methodology only for quantification of Simvastatin [25]. The analysis was carried out on an Agilent 1200 Series HPLC system (Agilent Technologies, Waldbronn, Germany) with a diode-array detector (λ = 238 nm). 20 μL samples were injected into a hypersil gold selectivity 5 μm, C${}_{18}$, (150 mm × 4.60 mm) column, with a pre-column guard cartridge (Thermo scientific, Rochester, NY, USA). Conditions of separation were: mobile phase of acetonitrile: water (70:30 *v*/*v*), flow rate of 1.2 mL/min and 25 ${}^{\circ }$C in an isocratic mode. Retention time for SIM was 4.8 min and for NIF was 2.2 min, respectively. The concentration of both APIs was determined from the area under the peak. A calibration curve in a range of 0.1–25 μg/mL was used.

### 2.7. Structural Analysis by FTIR

Structural analysis of crystalline SIM and NIF and co-amorphous binary system SIM–NIF (1:1) were carried out by infrared spectroscopy using a Fourier transform infrared spectrometer, Perkin-Elmer Spectrum 400 FTIR-ATR/NIR operating in the near-infrared region (Shelton, CT, USA). Samples were placed in contact with a horizontal attenuated total reflectance (ATR) accessory (Shelton, CT, USA). All spectra were scanned in the range of 380 to 4000 cm${}^{-1}$ with a resolution of 4 cm${}^{-1}$, and 16 scans were acquired and averaged. Spectrum™ software from Perkin-Elmer was used for analysis of spectra and the spectra were normalized and the baseline was corrected.

## 3. Results and Discussion

### 3.1. Thermal Characterization

**Figure 2:**
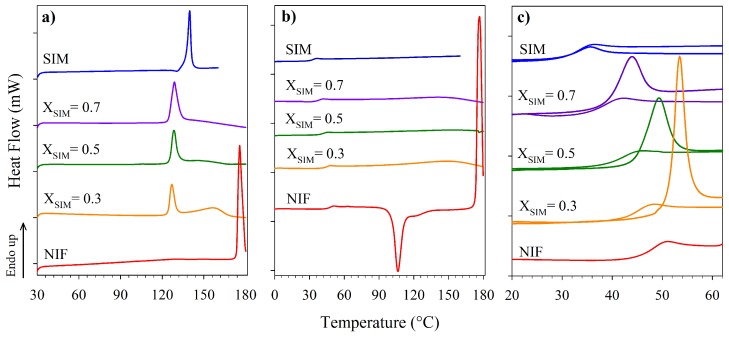
Thermograms for SIM, NIF, and representative binary mixtures (**a**) First heating of samples in crystalline phase. (**b**) Second heating of samples in amorphous form. (**c**) Glass transition temperatures of the amorphous samples freshly prepared and after one year of storage. Thermograms were obtained by diffential scanning calorimetry, DSC using a heating rate of 10 ${}^{\circ }$C/min. Molar fractions are in terms of SIM.

Figure 2 shows thermal characterization results performed by DSC to determine the phase transitions of the pure components, as well as the binary system SIM-NIF both in crystalline and amorphous forms. Melting temperatures (Tm) of pure APIs were identified as a sharp single endothermic peak (see Figure 2a), the specific onset values shown in Table 2 are in agreement with values reported in the literature for pure SIM and NIF. According to previous reports, the NIF polymorph with melting temperature around 174 ${}^{\circ }$C corresponds to polymorph A. For SIM a single crystalline form has been reported [25,26,27]. For the case of crystalline binary systems, which were prepared with different molar compositions, the first endothermic peak corresponds to the melting of the eutectic composition and the second small peak corresponds to the liquidus temperature. The heating curves shown in Figure 2b correspond to the amorphous samples in which a glass transition process can be identified. Only some representative results are shown in Figure 2 for better visualization. The complete set of experimental transition temperatures are summarized in Table 2.

The thermogram for amorphous NIF (Figure 2b), in addition to the glass transition, also shows an exothermic peak indicating crystallization, which means that this particular drug is thermally unstable in the amorphous state when reheated. In contrast, pure SIM and the co-amorphous systems proved to be stable in their amorphous state since none of them presented any sign of crystallization during reheating. With this result, it can be established that SIM is a good stabilizer for NIF. It is important to mention that NIF has been reported as very unstable API in the amorphous state even in the presence of surfactants and excipients [28,29]. A zoom in to the glass transition temperatures (Tg) of the amorphous samples prepared in situ is presented in Figure 2c, in which the thermograms for the amorphous materials measured after one year storage are also added. The glass transition processes in this case clearly show an overshoot resulting from the structural relaxation of the amorphous materials towards an equilibrium structure.

**Figure 3:**
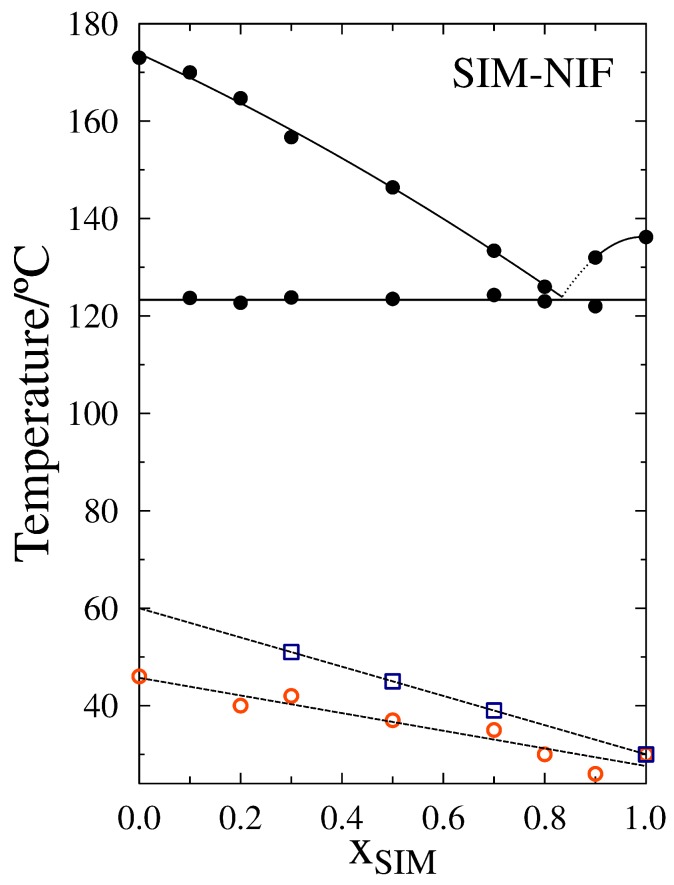
Phase transition diagram for the binary system SIM-NIF. Liquidus and eutectic temperatures shown in filled circles. In the phase diagram are also included the glass transition temperatures measured immediately after the quenching (empty circles) and after storage (empty squares).

With the phase transition temperatures obtained for the system SIM-NIF a phase diagram was built, shown in Figure 3. The data suggests that a eutectic composition exists close to X${}_{\mathrm{SIM}}=0.84$ with a eutectic temperature of 123 ${}^{\circ }$C. The values for the glass transitions of the binary system are also added to the phase diagram, both of the prepared in situ right after the melting and the ones measured after one year of storage. The values of glass transition decrease linearly with higher molar fractions of Simvastatin. There is just one prior observation reported of increments in Tg for amorphous drugs during shelf life at room temperature [17]. These findings confirm that measurements of glass transition should be made after enough time has passed as to reach a more relaxed glassy structure in order to have a more objective value of this transition.

### 3.2. Results of Stability of the Amorphous State by PXRD

**Figure 4:**
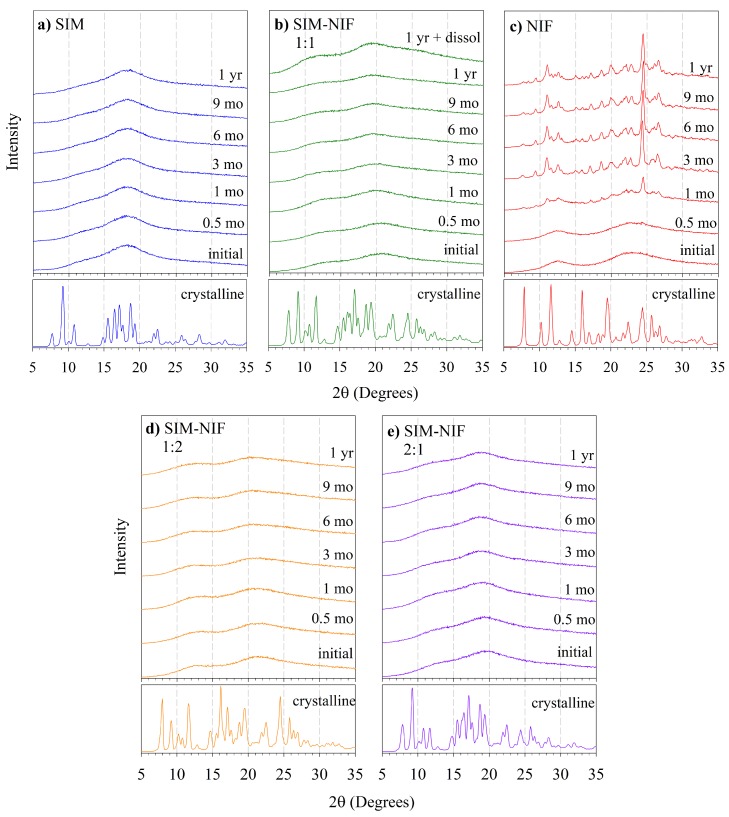
Powder X-ray diffraction pattern for samples in crystalline form and samples in amorphous form studied as a function of storage time, measured by X-ray diffraction (XRD). (**a**) Pure SIM (**b**) SIM-NIF 1:1 (**c**) Pure NIF. (**d**) SIM-NIF 1:2 (**e**) SIM-NIF 2:1 Also, the XRD pattern of a remainder of a sample subjected to a dissolution process is shown in (**b**).

In order to determine the stability of the amorphous state during storage time, all samples were analyzed by PXRD to monitor recrystallization. Crystalline and amorphous samples of pure APIs, physical mixtures of the two crystalline APIs of the components and the amorphous binary systems were measured. Figure 4a shows the diffraction pattern for crystalline SIM; diffraction peaks are in agreement with previous results, showing characteristic peaks at 9.3${}^{\circ }$, 10.8${}^{\circ }$, 17.2${}^{\circ }$, 18.7${}^{\circ }$, and 22.5${}^{\circ }$ for 2θ, which correspond to reported values [25]; it is the only stable polymorph at standard conditions [30]. The PXRD pattern for crystalline NIF is shown in Figure 4c, and the diffraction peaks coincide with the ones reported for the polymorph A [27], showing characteristic peaks at 7.9${}^{\circ }$, 10.3${}^{\circ }$, 11.6${}^{\circ }$, 16.0${}^{\circ }$, 24.5${}^{\circ }$ and 25.7${}^{\circ }$ for 2θ. For amorphous samples, as expected, there is a lack of short-range order and therefore the PXRD pattern becomes a broad band. Amorphous samples were stored at room temperature and monitored periodically. Pure vitreous or amorphous SIM remained stable in amorphous state throughout the evaluation time of one year, not showing any signs of recrystallization. Amorphous pure NIF showed crystallization before one month of storage and the intensity of the peaks grew with time (see Figure 4c). Partially recrystallized NIF showed characteristic peaks at 9.5${}^{\circ }$, 11.0${}^{\circ }$, 17.1${}^{\circ }$, 24.5${}^{\circ }$ and 26.7${}^{\circ }$ for 2θ, indicating recrystallization into polymorph C, which is in accordance to the results reported by Grooff et al. [31]. The PXRD pattern for recrystallized NIF sample after one year presented also weak peaks corresponding to the polymorph A.

The co-amorphous system SIM-NIF 1:1 did not show recrystallization for the evaluation time of one year, as shown in Figure 4b. It is important to emphasize that a significant stability of NIF against recrystallization was achieved with the preparation of the amorphous binary systems, in comparison to the pure API, including molar ratios 1:2 and 2:1 shown in Figure 4d,e, respectively.

An additional evidence of the stability in the amorphous state of SIM-NIF formulation during dissolution profile is shown also as a part of the XRD results of Figure 4b. Analysis on the remainder of a tablet after a dissolution profile experiment showed that the amorphous state was retained even when sample was exposed to 6 h of contact with the solvent. Although it is expected that water serves as a plasticizer, in this specific case this effect did not lower the physical stability of the sample. It is important to mention that this particular sample was first stored for one year and then exposed to dissolution process, confirming the high stability of the co-amorphous system.

### 3.3. Results of Dissolution Profiles

**Figure 5:**
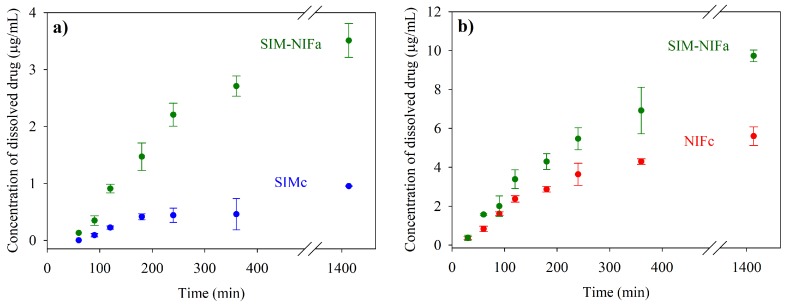
Aqueous dissolution profiles for pure crystalline APIs (SIMc and NIFc) and co-amorphous formulation SIM-NIF: (**a**) solubility for SIM (**b**) solubility for NIF.

**Figure 6:**
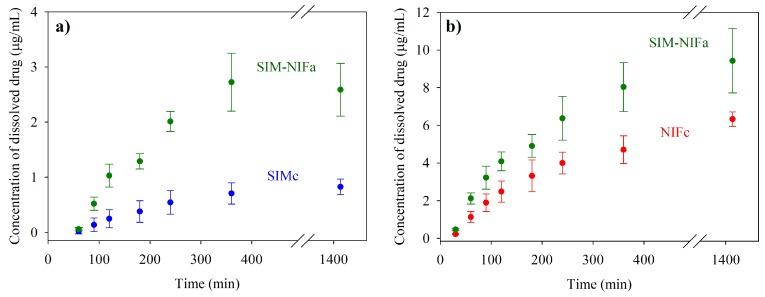
Dissolution profiles in USP Phosphate Buffer pH 6.8 for pure crystalline APIs (SIMc and NIFc) and co-amorphous formulation SIM-NIF: (**a**) solubility for SIM (**b**) solubility for NIF.

Dissolution profiles were evaluated to compare the solubility of pure APIs in their crystalline form with co-amorphous formulations. Figure 5 shows the results for the dissolution in water; as it can be observed, a significant increment of solubility, of 3.7 times, was achieved for Simvastatin. For SIM–NIF (1:1) formulation, besides the enhanced solubility of SIM also an increment of 1.7 times in NIF solubility was observed in this new amorphous formulation. Dissolution profile was also studied in a controlled pH medium using phosphate buffer (pH = 6.8). Increments in solubility were similar to the ones observed in water, being 3.1 and 1.5 times for SIM and NIF, correspondingly, in the co-amorphous system (see Figure 6).

The enhancement of solubility observed for SIM in the present study is a very important achievement considering the previous attempts of co-amorphous formulations (API–API) containing SIM in which no enhancement of solubility for this particular API was attained. In a previous formulation prepared with Simvastatin and Glipizide; improvement of Glipizide’s solubility was reported but no significant change in solubility for SIM was obtained [25]. Similarly, in a different work of a formulation of Simvastatin-Gliclazide, even though there was an increment of Gliclazide’s solubility, no increase for Simvastatin was observed [32].

**Figure 7:**
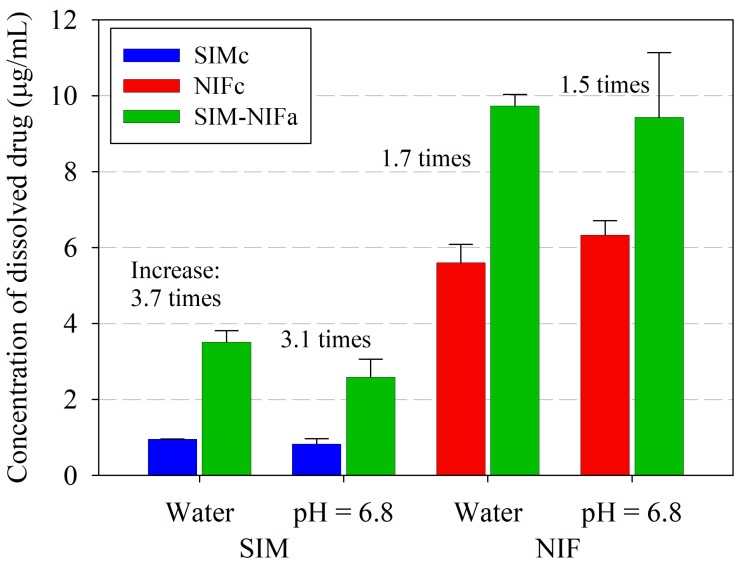
Comparison of maximum solubility values of pure APIs in their crystalline form (SIMc and NIFc) and co-amorphous formulation (SIM-NIF). Maximum solubility values shown were obtained in two different media: deionized water and a controlled pH medium using USP phosphate buffer (pH = 6.8).

In Figure 7a comparison of maximum solubilities for SIM and NIF in the different media studied are shown. Taking into account that an increment for nifedipine was achieved as well as for SIM, this makes the present formulation a promising alternative to be used in combination therapy. Maximum solubility values are shown in Table 3.

It is important to mention that, although there are different studies with either SIM or NIF, these formulations use sugars, amino acids or polymers as excipients [11,13,16]. Comparing the solubility of our system with these previous studies is not part of the discussion of the present work since the purpose of this paper is to make a contribution in the simultaneous treatment of high incidence diseases such as hypertension and hypercholesterolemia through combination therapy. The solubility enhancements of previous studies with these excipients may be used in synergy with the API–API system presented in this work in combined therapy.

### 3.4. Results of Structural Analysis by FTIR

**Figure 8:**
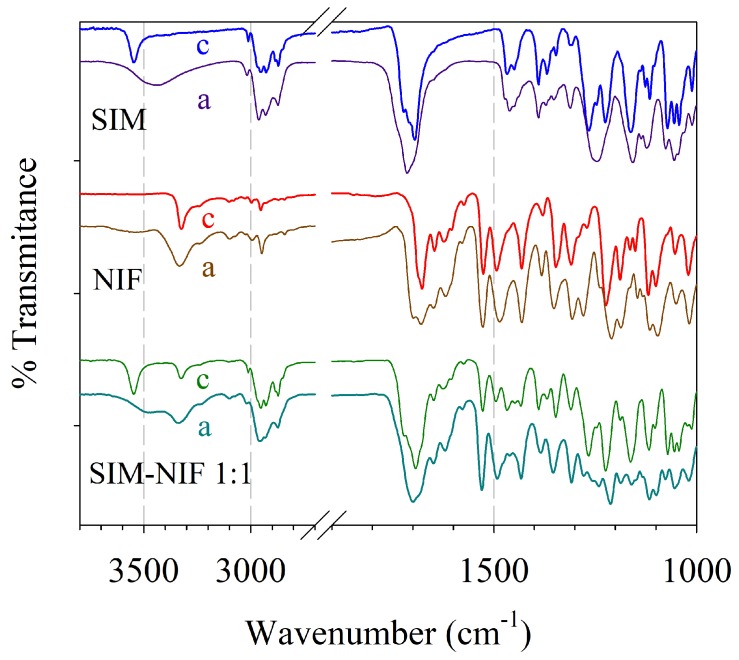
FTIR spectra of crystalline (c) and amorphous (a): SIM, NIF, and co-amorphous formulation SIM–NIF 1:1.

FTIR was used to analyze the possible formation of intermolecular interactions between SIM and NIF in the co-amorphous binary system. Figure 8 shows the infrared spectra of pure crystalline and amorphous APIs, and the 1:1 amorphous SIM–NIF system. For crystalline SIM, the signals found correspond to the most representative functional groups: at 3548 cm${}^{-1}$, assigned to the OH stretching; 2955 cm${}^{-1}$, for the methyl C–H asymmetric stretching; 1695 cm${}^{-1}$ for the carbonyl stretching (C=O) of the ester, and 1162 and 1115 cm${}^{-1}$ assigned to the C–O–C bond of the ester; all these signals agree with previous reports for SIM [25,33,34].

In the case of crystalline NIF, the most important signals are assigned to the N–H stretching, ester carbonyl C=O, –NO${}_{2}$ and C–C–O of the ester at 3324, 1678, 1309 and 1223 cm${}^{-1}$, respectively. As in the case of SIM, these signals agree with the values previously reported [17,24,35,36].

When comparing the pure crystalline materials against the amorphous samples, spectra show the characteristic broadening in the signals, due to the disorder in the molecules caused by the amorphization (Figure 8). Shifts in signals for functional groups reveal inter-molecular interactions of the hydrogen bond type. It was found that for SIM there were mainly two signals that presented shifts: the first one the stretching of the O–H from 3548 cm${}^{-1}$ to 3443 cm${}^{-1}$; this shift from higher to lower wavenumber was attributed to the formation of intermolecular interactions, mainly hydrogen bonds, between different O–H from a molecule of SIM. A shift from lower to higher wavenumber (1695 to 1714 cm${}^{-1}$) was observed for the stretching of the C=O of the ester, in agreement with a previous report [25]. Contrary to the observed shift for the –OH functional group, the increment in wavenumber for the carbonyl indicates the loss of participation of this functional group in the hydrogen bonding interactions of SIM after its amorphization [17,36].

In the case of amorphous NIF, similar results were reported by Martínez et al. [17] for NIF, since both the –NH and the C=O signal of the ester presented a shift from lower to higher wavenumber, from 3324 to 3334 cm${}^{-1}$ and from 1687 to 1698 cm${}^{-1}$, respectively indicating the loss of hydrogen bond interactions. This result is in agreement with nifedipine instability in the amorphous state and its tendency to crystallize as a function of time (see Figure 4) because of hydrogen-bonding ordering effect.

Figure 8 also shows the spectrum for the co-amorphous binary system. It is interesting to notice that the signal corresponding to the C=O stretching of the carbonyl of SIM appears at 1700 cm${}^{-1}$, compared to the pure amorphous SIM in which the shift is from higher to lower wavenumber (1714 to 1700 cm${}^{-1}$), almost returning to the position observed for crystalline Simvastatin (1695 cm${}^{-1}$). As previously described, during the amorphization of SIM, hydrogen bonds in which carbonyl groups are participating are lost by molecular disorder caused by amorphization.

In the case of signals corresponding to NIF, no shifts were observed when comparing the co-amorphous binary system with the pure amorphous material. Therefore, its stability in the amorphous state in the binary system cannot be related to the formation of intermolecular interactions; it can, however, be explained in terms of the presence of molecules of SIM provoking a mechanical hindrance [37], preventing the interactions between NIF molecules leading to its recrystallization, and thus the binary system to remain in the amorphous state for a longer time than pure amorphous nifedipine.

## 4. Conclusions

A new co-amorphous system with potential use in combination therapy of high incidence diseases such as hypertension and hypercholesterolemia was prepared and characterized. Results show that the formulations are stable in the amorphous state after long storage times (more than a year), during heating and also after 6 hours of dissolution process. This result is an important accomplishment since nifedipine has been reported as a very unstable API in the amorphous form. The increments in aqueous solubility, of 3.7 and 1.7 times for SIM and NIF, correspondingly, in the co-amorphous system, are significant as it is one of the few cases in which both APIs present an increment in solubility. The present study contributes to the efforts currently made in the health sector and the pharmaceutical industry since it provides a strategy for the combined treatment of common illnesses that currently affect the general population worldwide.

## Figures and Tables

**Table 1:** Principal active pharmaceutical ingredients (APIs) used in the treatment of hypertension (HT) and hypercholesterolemia (HCL) and their respective classification in the biopharmaceutical classification system (BCS).

API	Illnes	Solubility	Permeability	BCS	Reference
Pravastatin	HCL	High	High	I	[5]
Glimepiride	HCL	Low	High	II	[6]
Atorvastatin	HCL	Low	High	II	[7]
Simvastatin	HCL	Low	High	II	[5]
Fenofibrate	HCL	Low	High	II	[8]
Rosuvastatin	HCL	Low	High	II	[9]
Gemfibrozil	HCL	Low	High	II	[10]
Ezetimibe	HCL	Low	High	II	[11]
Metoprolol	HT	High	High	I	[6]
Telmisartan	HT	Low	High	II	[6]
Losartan	HT	High	Low	III	[12]
Irbesartan	HT	Low	High	II	[5]

**Table 2:** Experimental values for the eutectic (T${}_{\mathrm{eut}}$), liquidus temperature (Tm) and glass transition temperatures (Tg) for the pure components (SIM, NIF) and the binary systems. Molar fractions are reported in terms of SIM.

Sample	T${}_{\mathrm{eut}}$/${}^{\circ }$C	Tm/${}^{\circ }$C${}^{*}$	Tg/${}^{\circ }$C
SIM		136	30
X${}_{\mathrm{SIM}}=0.9$	122	132	26
X${}_{\mathrm{SIM}}=0.8$	123	126	30
X${}_{\mathrm{SIM}}=0.7$	124	133	35
X${}_{\mathrm{SIM}}=0.5$	124	146	37
X${}_{\mathrm{SIM}}=0.3$	124	157	42
X${}_{\mathrm{SIM}}=0.2$	123	165	40
X${}_{\mathrm{SIM}}=0.1$	124	170	42
NIF		174	46

* Tm corresponding to the liquidus temperature. Tg was measured at a heating rate of 10 °C/min.

**Table 3:** Experimental values for the concentration of the dissolved drug in deionized water and phosphate buffer pH 6.8. (n=3±s.d.)

Drug	Sample	Deionized Water	Phosphate Buffer pH = 6.8
		Concentration of Dissolved Drug at 24 h (μg/mL)	Concentration of Dissolved Drug at 24 h (μg/mL)
SIM	SIMc	0.95±0.01	0.82±0.14
	SIM–NIFa	3.51±0.30	2.59±0.48
NIF	NIFc	5.60±0.48	6.33±0.38
	SIM–NIFa	9.73±0.30	9.43±1.71

## Abbreviations

The following abbreviations are used in this manuscript:

ACS	American Chemical Society
AR (ACS)	American Chemical Society Commitee on Analytical Reagents
HT	Hypertension
HCL	Hypercholesterolemia
SIM	Simvastatin
NIF	Nifedipine
API	Active Pharmaceutical Principle
DSC	Differential Scanning Calorimetry
HPLC	High performance liquid chromatography
FTIR	Fourier transform infrared spectroscopy
PXRD	powder X-ray diffraction
Tg	Glass Transition Tempeature
Tm	Melting Temperature
USP	United States Pharmacopeia.
